# Role and mechanisms of exercise therapy in enhancing drug treatment for glioma: a review

**DOI:** 10.3389/fimmu.2025.1576283

**Published:** 2025-04-30

**Authors:** Guanghui Wu, Yisheng Chen, Chong Chen, Jianling Liu, Qiaowu Wu, Yazhen Zhang, Runqiong Chen, Jianzhong Xiao, Yusheng Su, Haojun Shi, Chunsheng Yu, Miao Wang, Yifan Ouyang, Airong Jiang, Zhengzhou Chen, Xiao Ye, Chengwan Shen, Aikebaier Reheman, Xianjun Li, Ming Liu, Jiancheng Shen

**Affiliations:** ^1^ Department of Neurosurgery, Ningde Clinical Medical College, Fujian Medical University, Ningde, Fujian, China; ^2^ Department of Neurosurgery, Ningde Municipal Hospital, Ningde Normal University, Ningde, Fujian, China; ^3^ Fujian Key Laboratory of Toxicant and Drug Toxicology, Medical College, Ningde Normal University, Ningde, Fujian, China; ^4^ Department of Neurosurgery, School of Medicine, Loma Linda University, Loma Linda, CA, United States; ^5^ Department of Physiology and Pharmacology, School of Medicine, Loma Linda University, Loma Linda, CA, United States; ^6^ Department of Neurosurgery and Anesthesiology, School of Medicine, Loma Linda University, Loma Linda, CA, United States; ^7^ NHC Key Laboratory of Diagnosis and Treatment on Brain Functional Diseases, The First Affiliated Hospital of Chongqing Medical University, Chongqing, China; ^8^ School of Physical Education, Ningde Normal University, Ningde, Fujian, China; ^9^ Faculty of Chinese Medicine and State Key Laboratory of Quality Research in Chinese Medicines, Macau University of Science and Technology, Macau, Macau SAR, China

**Keywords:** glioma, exercise therapy, drug treatment, immune system, blood-brain barrier

## Abstract

Gliomas, particularly glioblastoma (GBM), are among the most aggressive and challenging brain tumors to treat. Although current therapies such as chemotherapy, radiotherapy, and targeted treatments have extended patient survival to some extent, their efficacy remains limited and is often accompanied by severe side effects. In recent years, exercise therapy has gained increasing attention as an adjunctive treatment in clinical and research settings. Exercise not only improves patients’ physical function and cognitive abilities but may also enhance the efficacy of conventional drug treatments by modulating the immune system, suppressing inflammatory responses, and improving blood-brain barrier permeability. This review summarizes the potential mechanisms of exercise in glioma treatment, including enhancing immune surveillance through activation of natural killer (NK) cells and T cells, and increasing drug penetration by improving blood-brain barrier function. Additionally, studies suggest that exercise can synergize with chemotherapy and immunotherapy, improving treatment outcomes while reducing drug-related side effects. Although the application of exercise therapy in glioma patients is still in the exploratory phase, existing evidence indicates its significant clinical value as an adjunctive approach, with the potential to become a new standard in glioma treatment in the future.

## Graphical Abstract

Research on Exercise Therapy in Improving Drug Treatment for Glioma This figure illustrates the potential of exercise therapy to enhance glioma treatment. **(A)** Conventional therapies face limitations, such as drug resistance and side effects. **(B)** Glioma treatment is challenged by the blood-brain barrier, tumor microenvironment and immunosuppression. **(C)** Exercise modulates the immune response, reduces inflammation, and promotes tumor suppression via myokines. **(D)** Exercise enhances drug sensitivity and reduces chemotherapy resistance. **(E)** It improves physical endurance, cognitive function, neurological recovery and mental well-being. **(F)** Despite research gaps, integrating exercise into clinical oncology through personalized programs and multidisciplinary collaboration holds promise for the management of glioma.

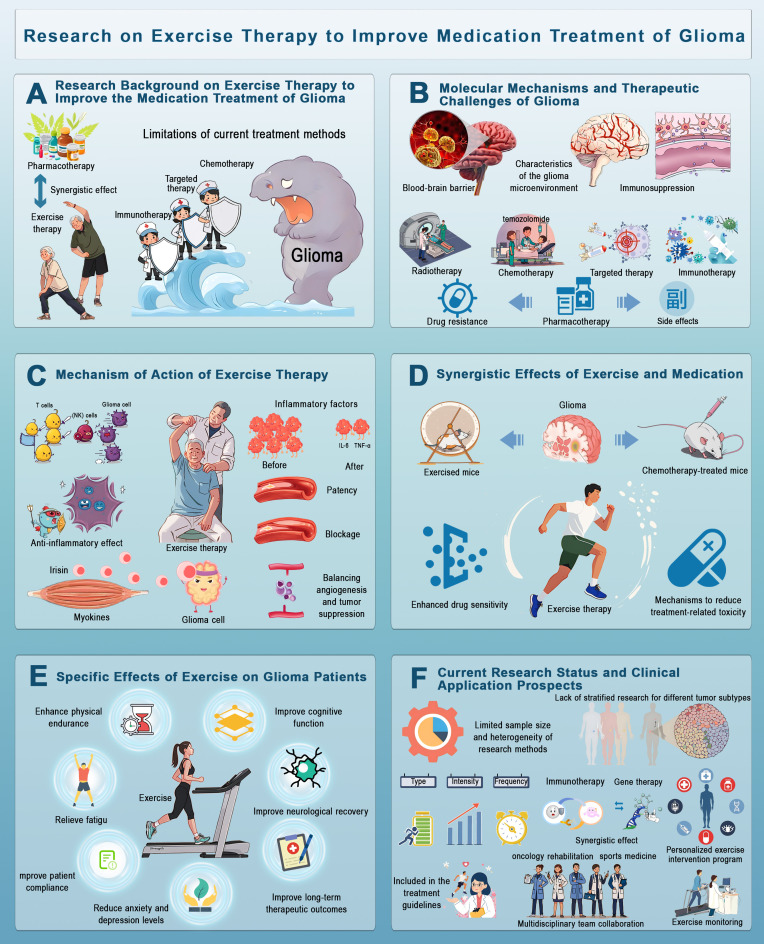

## 1 Introduction

### 1.1 Epidemiology and clinical burden

Gliomas, especially glioblastomas (GBM), are among the most common and aggressive brain tumors (1). With a high degree of malignancy and poor prognosis, current treatment options such as chemotherapy, targeted therapy, and immunotherapy have many limitations (2). Glioblastomas account for approximately 48% of adult brain malignancies, with standard treatment primarily relying on radiotherapy and temozolomide chemotherapy (3). However, these therapies have not significantly improved long-term survival and quality of life for patients (4). The biological characteristics of glioblastomas present numerous challenges during treatment. These tumors are characterized by dense vasculature and are often accompanied by vasogenic edema and mass effects, which exacerbate neurological symptoms and lead to a poor quality of life (5). Additionally, glioblastomas exhibit a highly immunosuppressive microenvironment, which further complicates treatment efforts. Although there have been significant advances in cancer treatment in recent years, therapeutic outcomes for gliomas remain limited, particularly in patients with high-grade gliomas (HGGs) (6). The primary treatment goal for patients with HGG is to achieve progression-free survival and delay cognitive and neurological decline as much as possible (7). For these patients, health-related quality of life (HRQOL) has become a critical measure for evaluating treatment effectiveness (8).

Although brain tumors account for a relatively small proportion of all malignancies (1. 4%), their negative impact on both physical and mental health is profound (9). Patients with brain tumors often suffer from functional impairments, experiencing not only physical dysfunction but also significant reductions in cognitive abilities and social psychological well-being. Consequently, cancer rehabilitation, particularly research focused on patients with brain tumors, has become a prominent area of study. With advancements in early diagnosis and treatment, the overall survival rate of patients with cancer has significantly improved (10). However, the challenge of maintaining quality of life after treatment remains severe for patients with gliomas. This is particularly important given the frequent occurrence of neurological deficits, fatigue, and cognitive decline that persist after conventional treatment. In this context, complementary therapies, such as exercise, may offer potential benefits by improving both physical function and cognitive outcomes.

The incidence of brain tumors increases with age, and survival rates decline with age at diagnosis. The median age at diagnosis of most brain tumors is 56 years. However, it is important to note that brain tumors remain one of the most common malignant cancers in children (11). The incidence of different types of brain tumors varies significantly across age groups. In children, embryonal/neuroectodermal tumors and pilocytic astrocytomas are more prevalent, whereas in adults, meningiomas and malignant gliomas are more common (12). The impact of brain tumors on patient health extends beyond physical effects and includes a decline in social and psychological functioning (13). Treatments such as surgery, radiotherapy, and chemotherapy not only cause direct physical damage but can also lead to long-term side effects, further impairing a patient’s ability to work and interact socially (14). Therefore, glioma treatment should focus not only on prolonging survival but also on enhancing post-treatment functional recovery and quality of life. Moreover, emerging evidence suggests that incorporating physical exercise during or after treatment could offer additional benefits by modulating the immune response, reducing inflammation, and improving drug delivery.

### 1.2 Burden of brain cancer and other central nervous system cancers

Although brain cancer and other central nervous system (CNS) tumors account for a relatively small proportion of all cancers, their disease burden on patients is extremely heavy. Glioma is the most common malignant brain tumor in adults, accounting for 80% of all primary brain cancers. According to statistics, the five-year relative survival rate for brain cancer is only 22%, which is significantly lower than that of other common cancers, such as breast cancer and prostate cancer, and much lower than the overall cancer survival rate (10, 15). Despite advancements in treatment, gliomas, especially glioblastomas, remain highly aggressive and have a poor prognosis, making them a major challenge for clinicians and researchers. Although treatment methods such as chemoradiotherapy (temozolomide) have somewhat improved patient survival rates, treatment-related side effects, particularly their impact on physical, cognitive, and social psychological functions, remain significant. These side effects persist throughout the treatment process and affect various stages of a patient’s life (16).

### 1.3 Potential of exercise as an adjunctive therapy

Ongoing research continues to unravel the intricate regulatory mechanisms through which physical activity modulates various biological processes. These findings are instrumental in advancing the refinement of intervention strategies and identification of novel therapeutic targets (16–18). In recent years, exercise has been increasingly recognized for its potential benefits as an adjunctive therapy for cancer patients. Research has shown that appropriate exercise interventions can significantly improve physical, social psychological, and cognitive functions in both healthy individuals and cancer patients (19). Exercise not only aids in the brain repair process in mice but also enhances cognitive abilities in both mice and humans (20). Although it remains unclear whether these benefits can be replicated in adult brain tumor patients undergoing treatment, animal studies have demonstrated the restorative effects of exercise on neurological function (21). In particular, the potential of exercise interventions in patients with brain tumors warrants further investigation.

A meta-analysis indicated that patients who engaged in physical activity after diagnosis had significantly higher disease-free survival and overall survival rates than those with the least physical activity (22). Furthermore, cancer-specific and all-cause mortality rates were reduced by 59% and 64%, respectively. This evidence highlights the importance of exercise as a potential therapeutic strategy for improving physical function, modulating immune responses, and enhancing overall treatment efficacy (23–25). The American College of Sports Medicine’s exercise prescription guidelines also provide strong evidence supporting the role of exercise interventions in managing anxiety, depressive symptoms, fatigue, quality of life (QoL), and physical function. Notably, exercise interventions have also shown benefits for bone health and sleep (26).

## 2 Molecular mechanisms of glioma and treatment challenges

### 2.1 Molecular and biological characteristics of glioma

**Figure 1:**
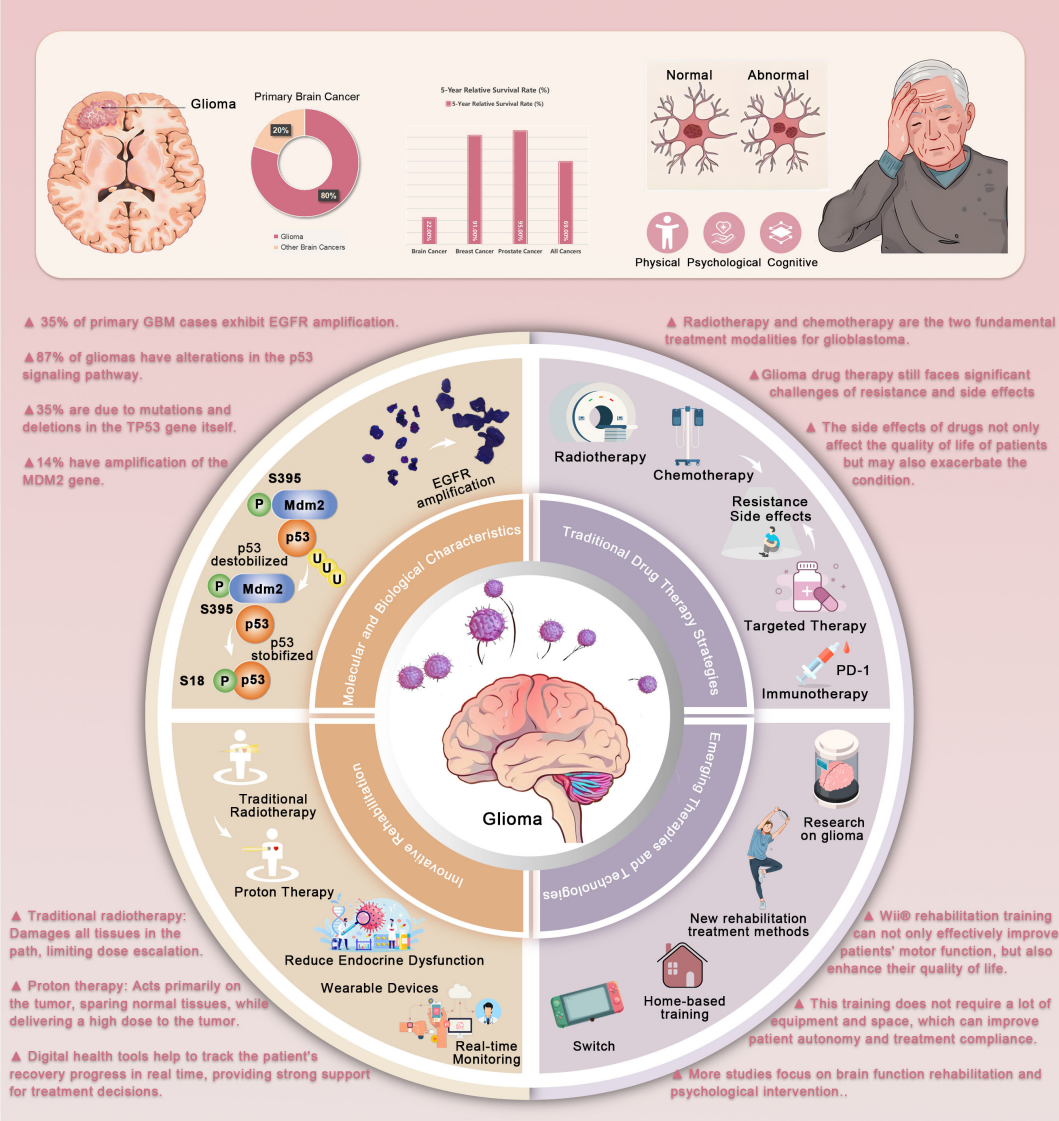
Molecular Characteristics, Treatment Strategies, and Emerging Technologies for Glioma Management. This figure outlines glioma pathophysiology, traditional treatments, and emerging therapies. The p53 pathway’s dysregulation, influenced by phosphorylation, is key in glioma progression. Traditional treatments, including radiotherapy and chemotherapy, face resistance and side effects. New approaches, like PD-1 inhibitors and immunotherapy, are being explored. Proton therapy offers more precise targeting of tumors, sparing healthy tissues. Rehabilitation technologies like home training and digital monitoring improve patient autonomy and compliance.

Glioma, particularly GBM, is a significant concern because of its high invasiveness, heterogeneity, and strong resistance to current treatments as shown in **Figure 1**. The heterogeneity of glioma cells is reflected not only in cell morphology but also in gene expression, epigenetic modifications, metabolic characteristics, and the complexity of the immune microenvironment (27). This heterogeneity contributes to the difficulty in effectively treating gliomas, as traditional therapies often fail to address all tumor subpopulations, leading to tumor recurrence (28). At the molecular level, gliomas are typically characterized by gene mutations, chromosomal aberrations, and epigenetic alterations (29). For instance, amplification of the EGFR gene and mutations in TP53 are common genetic features of glioblastoma. These mutations are closely associated with the high invasiveness, rapid growth, and treatment resistance observed in gliomas. Further studies have shown that EGFR amplification activates a series of oncogenic signaling pathways, such as the PI3K/AKT and Ras/MAPK pathways, promoting glioma growth, proliferation, and migration (30). Additionally, the methylation status of the MGMT (O-6-methylguanine-DNA methyltransferase) gene is closely linked to the response of patients with glioma to temozolomide (31). MGMT methylation suppresses its expression, enhancing patient sensitivity to chemotherapy drugs (31).

In addition to genetic mutations, the metabolic characteristics of glioma cells reflect their high heterogeneity. Studies have found that glioma cells often exhibit abnormal glucose metabolism, such as the phenomenon of “aerobic glycolysis” (the Warburg effect) (32). This metabolic process allows tumor cells to preferentially generate energy through glycolysis, even in the presence of sufficient oxygen (33). This not only provides the energy needed for tumor cell growth but also leads to the accumulation of lactate, altering the tumor microenvironment and promoting tumor invasiveness (34). The immune microenvironment of gliomas is also a crucial factor contributing to therapeutic resistance. Research indicates that glioma cells suppress antitumor immune responses through multiple mechanisms (35). For example, gliomas often upregulate immune checkpoint molecules, such as PD-L1, inhibiting T-cell immune responses (36). Furthermore, the infiltration of tumor-associated macrophages (TAMs) and other immune-suppressive cell populations is one of the reasons for glioma’s resistance to treatment (37). This immunosuppressive microenvironment not only shields tumor cells from immune surveillance but also fosters tumor progression and metastasis (38).Investigating the intricate interplay of biomolecular mechanisms in gliomas-including signaling pathways activated by genetic mutations, metabolic reprogramming, and immune microenvironment regulation – enables comprehensive elucidation of tumorigenic processes, thereby informing strategic development of targeted therapeutic agents (39, 40).

### 2.2 Traditional and emerging pharmacological treatment strategies

#### 2.2.1 Radiotherapy and chemotherapy

Radiotherapy and chemotherapy are the two mainstay treatments for glioblastoma. Despite their essential role in clinical treatment, their effectiveness is often limited (41). Radiotherapy kills tumor cells through high-energy radiation but is also challenging to target solely at tumor cells, often causing damage to normal brain tissue (42). This is particularly problematic in pediatric patients, in whom long-term cognitive dysfunction caused by radiotherapy remains a serious side effect. In addition to cognitive damage, radiotherapy may lead to brain atrophy, endocrine dysfunction, and other neurological impairments (43). To reduce these side effects, increasing attention is being paid to more precise radiotherapy techniques, such as stereotactic radiotherapy (SRT) and proton beam radiotherapy (PBRT), which enhance targeting accuracy and reduce damage to healthy tissue (44). Temozolomide (TMZ) is a standard drug used in the treatment of glioblastoma. It kills tumor cells by interfering with the DNA repair processes. However, due to the resistance of tumor cells, the efficacy of temozolomide is often limited (45). The resistance mechanisms may involve the expression levels of the MGMT gene, changes in the tumor microenvironment, and autophagy (46). To address this issue, researchers are exploring combination therapies with temozolomide to overcome tumor resistance and enhance the therapeutic efficacy.

#### 2.2.2 Targeted therapy and immunotherapy

In recent years, targeted therapy and immunotherapy have become popular topics in glioma research. Targeted therapy inhibits specific molecular targets within tumor cells to block their growth. For instance, targeted drugs against EGFR mutations and VEGF antibodies have been used in glioma treatment (47). However, owing to the molecular heterogeneity of gliomas, single-target drugs often fail to address the tumor’s diversity and resistance. In addition to single-target therapies, combination therapies targeting multiple pathways are being explored (28). Bioinformatics technologies have played a pivotal role in unraveling gene expression and regulatory mechanisms in gliomas, providing critical insights into the molecular basis of tumorigenesis and progression (48–50).Immunotherapy represents a revolutionary advancement in cancer treatment. By utilizing immune checkpoint inhibitors, the immune system is activated to enhance its recognition and destruction of tumor cells (51). Immunotherapy has provided new hope for patients with gliomas. However, the immune evasion mechanisms of gliomas render immunotherapy less effective than in other cancer types. The highly immunosuppressive microenvironment of gliomas and the frequent upregulation of immune checkpoints are major obstacles to the success of immunotherapy (52). Therefore, overcoming immune evasion mechanisms and improving the effectiveness of immunotherapy remain key challenges in the treatment of gliomas. In glioma immunotherapy, integrating bioinformatics with experimental approaches, such as epigenetic profiling, metabolic regulation analysis, and intercellular communication studies, uncovers novel immune cell mechanisms, thereby informing the optimization of therapeutic strategies (38, 53).

#### 2.2.3 Drug resistance and side effects

Despite continuous advancements in treatment strategies, drug resistance and side effects remain significant challenges in the treatment of gliomas. Tumor cell resistance to current treatments is not only closely linked to genetic mutations but also involves factors such as the tumor microenvironment, drug metabolism pathways, and the efficacy of the blood-brain barrier (54). The tumor microenvironment plays a crucial role in facilitating drug resistance by creating physical, biochemical, and immune barriers (55). For instance, overcoming the blood-brain barrier (BBB) for drug delivery in glioma requires interdisciplinary approaches that integrate pharmaceutics, materials science, and bioinformatics (38). Long-term treatment side effects are also a dilemma for patients with glioma. The side effects of drugs not only affect patients’ quality of life but can also exacerbate their conditions. For instance, while dexamethasone can alleviate brain edema and increased intracranial pressure in patients with glioma, prolonged use may lead to a range of side effects, such as osteoporosis, diabetes, and muscle atrophy (56). These chronic side effects can significantly reduce the patient’s functional capacity and quality of life, further complicating glioma management. These side effects highlight the need for more individualized treatment strategies to minimize the negative impact on patients’ health.

### 2.3 Emerging treatment methods and technologies

With technological advancements, emerging treatment methods and technologies have brought new hope for glioma treatment. Proton beam radiotherapy (PBRT) is a precise radiotherapy technique that offers greater targeting accuracy and fewer side effects than traditional photon radiotherapy (XRT). Proton beam radiotherapy can precisely focus radiation on the tumor site, minimizing radiation exposure to surrounding healthy tissues, and thus significantly reducing the risk of neurocognitive damage (57). Although large-scale clinical trial data remain limited, existing studies have shown that proton beam radiotherapy offers clear advantages in glioma treatment, particularly in reducing endocrine dysfunction and lowering the risk of tumor recurrence (58). In addition, the application of digital health technologies is gradually gaining attention in glioma treatment. By using wearable devices to monitor patients’ physiological data in real time, physicians can better understand the patient’s condition and adjust the treatment plan accordingly. These devices can monitor indicators such as activity levels, heart rate, and blood oxygen levels, and can also assess patients’ mobility and cognitive function (59). Meanwhile, AI-empowered CADD has advanced to high-throughput multi-scale simulations, providing intelligent frameworks for glioma-targeted therapies. Nanodelivery systems (NDS) optimize stem cell niches and enhance targeted delivery, while synergizing with exercise therapy to improve drug penetration and immune activation, thus overcoming blood-brain barrier and immunosuppressive challenges (60–62).Glioma research has established an integrated framework combining bioinformatics, advanced imaging technologies, and single-cell transcriptomics to systematically elucidate pathological mechanisms and white matter repair processes. The single-cell transcriptomic analysis methodology, developed based on references (63, 64), employs single-cell sequencing and high-dimensional omics technologies to characterize disease pathogenesis and cellular/molecular features within tumor immune microenvironments, offering novel perspectives for understanding disease progression and therapeutic responses (65–67). The synergistic application of bioinformatic tools and single-cell sequencing has enabled researchers to identify microenvironment-specific immune reaction patterns, providing empirical support for personalized treatment strategies (68, 69). These technological advancements have facilitated the implementation of multi-omics approaches (transcriptomics, metabolomics, proteomics) in clinical sample analysis, revealing disease-associated molecular signatures that inform early diagnosis and precision medicine (70, 71).

Clinical implementations demonstrate that CT-based multitask deep learning models can predict tumor-stroma ratios and treatment outcomes, while machine learning algorithms effectively identify lymph node metastasis patterns in gliomas, establishing evidence-based foundations for individualized therapy (72–76).Digital health monitoring systems significantly enhance rehabilitation management through real-time progression tracking (77). The integration of big data analytics and bioinformatics has become pivotal in glioma biomarker discovery and prognostic evaluation, with transcriptomic data mining enabling precise identification of critical genes and signaling pathways that inform drug development and early diagnostic markers (78–80). Cutting-edge approaches combining multi-omics integration, advanced bioinformatic analysis, and nanotechnology have elucidated potential therapeutic mechanisms of various agents (81, 82). Current investigations in glioma and other malignancies focus on extracellular vesicle (EV) applications as drug delivery systems through combined cellular biology and immune microenvironment analysis, creating novel paradigms for therapeutic innovation and prognostic assessment (83, 84).

## 3 Mechanisms of exercise therapy

### 3.1 Systemic antitumor effects

#### 3.1.1 The broad role of exercise in antitumor therapy

**Figure 2:**
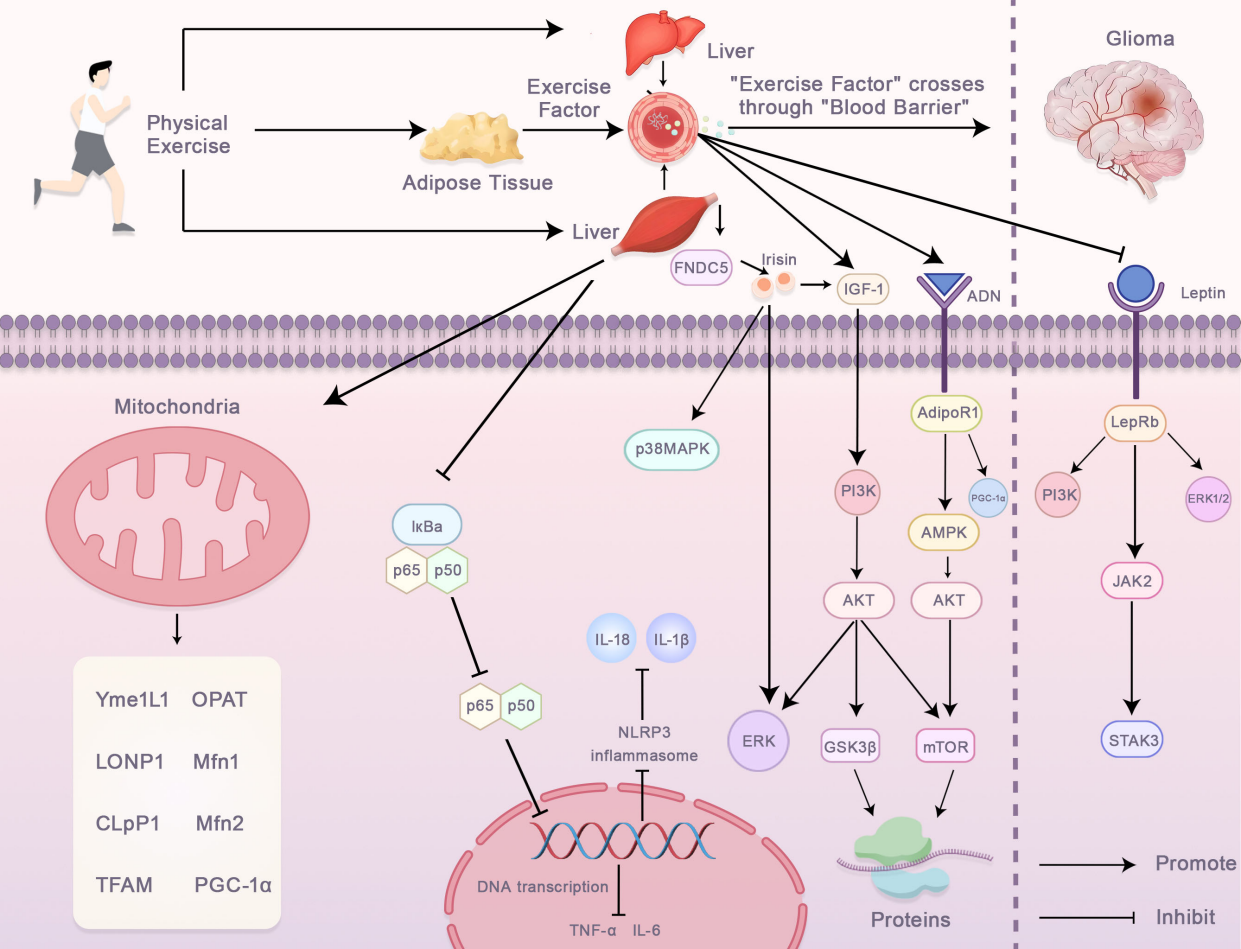
Molecular Mechanisms Underlying the Effects of Exercise on Glioma Progression. This figure shows how physical exercise influences glioma progression through systemic and intracellular pathways. Exercise releases factors like FNDC5, Irisin, IGF-1, and adiponectin from adipose tissue and the liver, which cross the blood-brain barrier and regulate glioma cells. These factors activate pathways that enhance mitochondrial function, reduce inflammation, and regulate glioma signaling. Exercise activates p38MAPK, inhibiting the NLRP3 inflammasome and lowering pro-inflammatory cytokines. IGF-1 and adiponectin activate survival pathways like PI3K/AKT, ERK, and GSK3β, promoting cellular homeostasis. Leptin also regulates glioma progression through the PI3K/JAK2/STAT3 axis. These adaptations suggest exercise therapy could complement glioma treatment.

Accumulating evidence underscores the capacity of exercise to modulate diverse biological processes, thereby facilitating the optimization of intervention strategies and identification of novel therapeutic targets (16–18). In recent years, exercise, as a natural health behavior, has been increasingly demonstrated to have potential in antitumor therapy (85). Exercise not only helps improve physical fitness, enhances cardiovascular function, and boosts the immune system, but also provides active support against tumor occurrence, progression, and recurrence through various mechanisms (86). This effect is particularly prominent in the treatment of highly malignant brain tumors, such as GBM. Owing to the limitations imposed by drug resistance, the BBB, and adverse factors in the tumor microenvironment, exercise as a non-pharmacological therapy has garnered significant attention for its potential antitumor effects (87). The antitumor effects of exercise can operate through various mechanisms, particularly by modulating the immune system, exerting anti-inflammatory effects, and improving the tumor microenvironment, thereby enhancing the body’s resistance to tumors (88). Studies have shown that moderate exercise can significantly improve immune system function by increasing the activity of immune cells, thereby enhancing the body’s ability to recognize and eliminate tumors as shown in **Figure 2** (89).

#### 3.1.2 Modulation of the immune system by exercise

The impact of exercise on the immune system is considered a core mechanism underlying its antitumor effects. The immune system plays a crucial role in tumor initiation and development (90). Tumor cells often evade detection by the host immune system through various mechanisms, whereas exercise enhances immune system function through multiple pathways, making it an effective strategy for preventing tumor progression (91). Studies have shown that moderate exercise enhances immune surveillance by regulating the quantity and activity of natural killer (NK) cells (25). NK cells are key components of the innate immune system, serving as the body’s first line of defense by effectively recognizing and eliminating tumor cells (92). By increasing the number and activity of these cells, exercise promotes immune system surveillance and attacks tumors (93). Furthermore, exercise can regulate T cell function, especially by enhancing the effectiveness of cytotoxic T cells, thereby promoting the clearance of tumor cells (94). Cytotoxic T cells recognize and kill tumor cells, assisting the body in eliminating tumors. By enhancing the function of these immune cells, exercise helps lower the incidence of tumors and slow their progression (50).

In addition to modulating T cells and NK cells, exercise regulates the expression of immunosuppressive factors (95). In the tumor microenvironment, immunosuppressive factors (such as IL-10 and TGF-β) often suppress immune cell function, allowing tumor cells to escape immune system attack (96). Research indicates that Exercise can lower the levels of these immunosuppressive factors, thereby enhancing the immune system’s ability to eliminate tumors (97). Chronic inflammation plays a key role in the occurrence and progression of tumors, and chronic inflammation in the tumor microenvironment is considered one of the primary factors promoting tumor growth and metastasis (98). Pro-inflammatory factors activate tumor cell proliferation, migration, and metastasis (99). Exercise, by reducing the expression of these pro-inflammatory factors, contributes to a less favorable environment for tumor growth and spread (100). The temporal dynamics of exercise-induced immune modulation are critical in this regard. Intermittent high-intensity interval training (HIIT) induces acute increases in cytotoxic T-cell infiltration within 24 h, whereas sustained moderate exercise promotes macrophage polarization toward the anti-tumor M1 phenotype. In glioma-bearing mice, HIIT reduced tumor volume by 25% compared to that in sedentary controls, whereas continuous exercise primarily improved survival rates. Clinical protocols should balance intensity and duration based on the treatment phase.

#### 3.1.3 Regulation of inflammatory response by exercise

Exercise is widely regarded as an effective anti-inflammatory agent, capable of modulating the immune system through various mechanisms and reducing the release of pro-inflammatory factors, thereby inhibiting tumor growth and metastasis (101). Studies have found that moderate exercise promotes the production of anti-inflammatory factors such as IL-10 and TGF-β (102, 103). These anti-inflammatory factors downregulate the expression of pro-inflammatory factors, thereby reducing chronic inflammation. For example, IL-10 inhibits the activation of T cells and macrophages, reducing inflammation, whereas TGF-β reduces immune cell activity, helping restore immune tolerance (104). Exercise can directly lower the expression of proinflammatory factors by modulating cellular signaling pathways. IL-6 and TNF-α, which are closely associated with chronic inflammation, are significantly reduced by exercise, alleviating inflammation in the tumor microenvironment (105). By reducing the release of pro-inflammatory factors, exercise can effectively inhibit tumor cell proliferation, migration, and metastasis, further suppressing tumor growth (106). Studies have shown that exercise boosts the activity of immune cells, such as T cells, NK cells, and macrophages, helping the immune system to more effectively recognize and eliminate tumor cells (101). Enhanced immune function not only directly combats tumors but also reduces the impact of chronic inflammation, which supports tumor growth (107).

#### 3.1.4 The potential of exercise in cancer immunotherapy

As immunotherapy emerges as a promising approach in cancer treatment, the immune-regulating effects of exercise have gained widespread attention. Immunotherapies, such as immune checkpoint inhibitors and CAR-T cell therapy, have shown significant efficacy against various tumor types (108). However, the effectiveness of immunotherapy is often hindered by immunosuppressive and pro-inflammatory factors in the tumor microenvironment. As a natural immune regulator, exercise can enhance immune cell activity, promote antitumor immune responses, and improve the effectiveness of immunotherapy (109). For instance, research has shown that exercise enhances the function of NK and T cells, enabling them to more effectively recognize and kill tumor cells (93). Exercise also improves blood supply in the tumor microenvironment, boosting immune cell infiltration in the tumor and enhancing the effects of immunotherapy (110).

#### 3.1.5 Exercise and immune checkpoint blockade

Immune checkpoint blockade (ICB) therapy, which includes inhibitors targeting PD-1, PD-L1, and CTLA-4, has revolutionized cancer treatment by reactivating the immune response against tumor cells. However, the clinical effectiveness of immune checkpoint inhibitors (ICIs) is often limited by the immunosuppressive tumor microenvironment (TME), which dampens the immune system’s ability to recognize and attack cancer cells. Exercise has emerged as a promising strategy to enhance the efficacy of immune checkpoint blockade therapy by modulating the TME and boosting immune cell function. Exercise can significantly improve immune surveillance by increasing the activity of immune cells, such as natural killer (NK) cells, cytotoxic T lymphocytes (CTLs), and dendritic cells (DCs). These immune cells are crucial for the recognition and elimination of tumor cells. Research has shown that exercise-induced systemic inflammation, characterized by increased cytokine release, can enhance the recruitment of immune cells to the tumor site, thereby sensitizing tumors to ICB therapy (25). Moreover, exercise reduces immune suppression in the TME, which often arises from the accumulation of regulatory T cells (Tregs) and myeloid-derived suppressor cells (MDSCs). By inhibiting these immunosuppressive cells, exercise helps enhance the antitumor immune response, making tumors more responsive to immune checkpoint inhibitors.

#### 3.1.6 Exercise’s role in systemic inflammation regulation

Exercise not only affects local inflammation but also regulates systemic inflammation, improving the overall immune status. Epidemiological studies have shown that individuals who engage in regular exercise have generally lower levels of chronic inflammation, which may be one of the key reasons for their lower cancer incidence (111). Moderate exercise reduces systemic inflammation by improving blood circulation, metabolism, and immune response, thereby lowering the risk of tumor occurrence (112). Chronic inflammation is a risk factor for various types of cancer, especially colorectal and breast cancers (111). Exercise significantly reduces systemic inflammation by improving blood circulation, modulating immune responses, and promoting metabolism, thereby supporting cancer prevention (86).

#### 3.1.7 Exercise’s role in inhibiting tumor metastasis

Tumor metastasis is often accompanied by intensification of local inflammation, with high levels of pro-inflammatory factors stimulating the invasiveness of tumor cells (98). Exercise can effectively inhibit the invasiveness of tumor cells by reducing local inflammation, thereby lowering the risk of metastasis (106). Studies have shown that exercise can slow tumor metastasis by reducing the levels of proinflammatory factors, such as IL-6 and TNF-α. Moreover, exercise improves endothelial function, inhibits tumor angiogenesis, and reduces the chances of tumor cell metastasis through the blood or lymphatic systems. Numerous animal experiments and clinical studies have demonstrated that regular exercise significantly lowers the risk of tumor metastasis and delays tumor progression (88).

#### 3.1.8 Synergistic effects of exercise and antitumor drugs

In addition to directly improving the tumor microenvironment and reducing inflammation, exercise has synergistic effects with traditional antitumor treatments, such as chemotherapy and radiotherapy. Research indicates that moderate exercise enhances the efficacy of chemotherapy drugs while reducing side effects (113). Exercise improves immune system function and reduces inflammation, helping chemotherapy drugs exert greater efficacy within the tumor microenvironment (24, 114). Specifically, exercise improves blood circulation, enhances drug delivery efficiency, and alleviates immune suppression in the tumor microenvironment, allowing chemotherapy drugs to target tumor cells more effectively (90, 110). Research also shows that exercise enhances the effects of immunotherapy, improving immune recognition and the elimination of tumor cells (50, 115).

#### 3.1.9 Clinical studies on the anti-inflammatory effects of exercise

Numerous clinical studies have validated the anti-inflammatory effects of exercise in reducing tumor-related inflammation (90). For instance, breast cancer patients undergoing chemotherapy demonstrated that regular low-intensity exercise significantly reduced the levels of inflammatory factors in the blood and improved their quality of life (116). Similar results have been confirmed in studies on colorectal cancer and other tumor types, demonstrating the potential of exercise in clinical cancer treatment (50, 117).

### 3.2 The remodeling of the tumor microenvironment by Exercise

#### 3.2.1 Altering immune and inflammatory factors in the tumor microenvironment

The TME plays a critical role in tumor growth, metastasis, and resistance to treatment. Exercise can effectively inhibit tumor progression by altering the levels of immune and inflammatory factors in the tumor microenvironment, particularly in the treatment of GBM (118). Moderate exercise can improve the tumor microenvironment by increasing NK and T cell infiltration, reducing the expression of immunosuppressive factors, and enhancing the effectiveness of immunotherapy (118).

#### 3.2.2 Improving blood-brain barrier permeability

The BBB is a complex structure composed of brain endothelial cells, basal membrane, astrocytes, and other cells that serves as a highly selective barrier to protect the brain from harmful substances. This barrier prevents the entry of pathogens, toxins, and other potentially harmful substances while limiting the effective delivery of many drugs, especially antitumor drugs. The BBB ensures the stability of the brain microenvironment and maintains neuronal function (119). However, its protective role complicates drug delivery in cancer treatments, particularly for invasive brain tumors such as glioblastoma, where the BBB becomes a major obstacle to treatment (120). In tumor therapy, especially for brain tumors, the ability to effectively penetrate the BBB and deliver drugs to the tumor site is crucial for improving treatment efficacy (121). Although various methods to enhance drug penetration through the BBB have been proposed, such as nanocarriers and drug delivery systems, these approaches often have limitations or potential side effects (122). Therefore, exploring natural physiological methods to improve BBB permeability has become an important area of research. Increasing evidence suggests that exercise, particularly regular aerobic exercise, may be a natural and effective way to improve BBB permeability (123, 124). Exercise, as a physical activity, influences multiple functions of blood circulation, the immune system, and the nervous system, and it has been increasingly shown to play a vital role in enhancing brain health. Specifically, exercise can improve BBB function through a series of complex physiological reactions, enhancing the permeability of the BBB to therapeutic drugs, thereby increasing the efficacy of antitumor medications in the brain (25, 123).

Studies have shown that regular aerobic exercise can improve endothelial cell function in the BBB through several mechanisms. Endothelial cells are the basic building blocks of the BBB and form tight junctions that control the selective permeability of substances. Exercise improves endothelial cell blood supply, promotes angiogenesis, and regulates the expression of molecules involved in cell-cell tight junctions, thereby increasing BBB permeability (123). Specifically, exercise has been shown to increase the levels of certain molecules in the blood, such as vascular endothelial growth factor (VEGF), matrix metalloproteinases (MMPs), and adrenomedullin, which can promote the “opening” of the BBB, making it easier for drugs to cross into the brain, thus enhancing drug efficacy (125). Exercise enhances BBB permeability through dual mechanisms (1): upregulation of vascular endothelial growth factor (VEGF) and erythropoietin (EPO), which promote endothelial cell proliferation and transiently loosen tight junction proteins (e.g., claudin-5, occluding); and (2) inhibition of matrix metalloproteinase-9 (MMP-9), which reduces the degradation of the basement membrane. Pharmacological agents targeting these pathways (e.g., anti-VEGF monoclonal antibodies) partially mimic the effects of exercise but lack systemic anti-inflammatory benefits. Notably, animal studies have demonstrated that voluntary running increases temozolomide penetration by 40% in orthotopic glioma models. Exercise-induced VEGF activates the PI3K/Akt signaling pathway in endothelial cells, promoting angiogenesis and transiently increasing BBB permeability. Concurrently, exercise reduces oxidative stress by upregulating antioxidant enzymes (e.g., SOD and Gaps), thereby stabilizing BBB integrity. Pharmacological agents mimicking these effects, such as anti-VEGF monoclonal antibodies (e.g., bevacizumab), show partial efficacy but lack the systemic benefits of exercise. Moreover, exercise can regulate neuroplasticity in the brain (126). Neuroplasticity is the brain’s ability to adapt to external changes, and exercise promotes neuroplasticity, which strengthens the interaction between neurons and endothelial cells, further improving BBB function (127). Notably, after prolonged exercise training, studies have shown enhanced blood supply to the brain and significant improvement in BBB permeability (123). This means that exercise not only improves drug delivery by enhancing blood circulation but also facilitates drug penetration by improving the adaptability of neural structures (128).

In addition to its direct effects on endothelial cell function, exercise enhances antioxidant capacity and reduces systemic inflammation, thereby reducing BBB damage (123). This mechanism is particularly important in brain tumor treatment, as the tumor microenvironment is often accompanied by significant inflammatory responses, which not only promote tumor cell growth but also damage the BBB (129). Therefore, exercise can alleviate these negative impacts through its anti-inflammatory and antioxidant effects, improving BBB function and enhancing drug efficacy (90, 130). These effects of exercise have been confirmed in animal studies and clinical research. In animal experiments, regular aerobic exercise (such as running and swimming) has been shown to significantly increase the permeability of antitumor drugs in the brain (131). In some studies, after several weeks of exercise training, the size of brain tumors in experimental animals was significantly reduced, and the concentration of drugs in the tumor region was notably higher (132). Although these studies are still mostly in the experimental phase, their potential has attracted widespread attention in the scientific community. Furthermore, exercise can enhance drug penetration by regulating molecular pathways related to the BBB (123). For instance, exercise increases ATP production, activating critical signaling pathways, such as the PI3K/Akt, MAPK, and NF-κB pathways, all of which play important roles in maintaining BBB integrity (133, 134). Exercise also regulates the function of cell adhesion molecules (such as tight junction proteins like ZO-1, occluding, and claudins) and transporters (e.g., P-glycoprotein), thereby modifying the selective permeability of the BBB (123). Through these mechanisms, exercise enables drugs that would typically struggle to penetrate the BBB, especially chemotherapeutic and immunotherapeutic drugs targeting tumors, to be delivered more effectively to the tumor site. It should be noted that the effect of exercise on BBB permeability may vary depending on the type, intensity, and duration of the exercise (123). Excessive and intense exercise may induce excessive stress responses in the body, leading to adverse effects (135). Therefore, moderate and consistent exercise is considered the optimal approach for improving BBB function (123). Many studies recommend engaging in at least three to four sessions of moderate-intensity aerobic exercise per week, with each session lasting more than 30 min, to achieve the best results (136). For patients with brain tumors, a personalized exercise plan can improve treatment outcomes and reduce side effects (137).

In summary, exercise improves BBB function through multiple pathways, enhancing the permeability of antitumor drugs and thus improving the efficacy of brain tumor treatments (138). Although most current studies are still in the animal or preclinical stage, the findings provide valuable insights into the potential of exercise as an adjunctive therapeutic approach for patients with cancer. In the future, with further clinical research, exercise may become an essential part of treatment regimens for patients with brain tumors, helping to enhance treatment outcomes and improve the quality of life. However, when exercise is used as an adjunctive therapy, individualized exercise plans must be developed based on the patient’s physical condition and treatment needs, and the plans should be implemented under the guidance of a professional medical team to ensure safety and effectiveness (139).

#### 3.2.3 Improving tumor blood supply and angiogenesis

Tumor growth and expansion largely depend on blood supply. As tumors continue to grow, their demand for oxygen and nutrients gradually increases, forcing tumor cells to secrete various growth factors, such as vascular endothelial growth factor (VEGF), to stimulate angiogenesis and ensure an adequate supply of nutrients and oxygen (140). Angiogenesis is not only a critical mechanism in tumor growth and metastasis but also directly impacts the tumor’s response to drug therapy (141). Tumor blood vessels are often structurally abnormal and functionally incomplete, resulting in an uneven blood supply and sometimes regional hypoxia (142). This promotes tumor cell metabolism, proliferation and dissemination. To overcome this inadequate blood supply, scientists have proposed strategies to enhance tumor treatment effectiveness by improving angiogenesis and blood supply within the tumor microenvironment (50, 143). Exercise, as a natural physiological modulator, has been shown to significantly influences tumor blood supply and angiogenesis (144). Although exercise enhances VEGF-mediated vascular normalization, it does not promote pathological angiogenesis. In GBM models, exercise reduced hypoxia-inducible factor-1α (HIF-1α) expression, thereby inhibiting aberrant vessel formation and improving perfusion for drug delivery.

The effect of exercise on tumor blood supply and angiogenesis primarily occurs by regulating various physiological responses in the body (145). Exercise enhances overall blood circulation, improving blood flow and oxygen transport, thereby increasing blood supply to the tumor region (146). This process serves a dual role in cancer therapy: on one hand, it provides more oxygen and nutrients, supporting tumor cell metabolism and growth; on the other hand, it enhances the delivery of antitumor drugs, enabling them to reach the tumor tissue more effectively and exert therapeutic effects (147). Additionally, exercise regulates local blood flow, preventing blood stagnation and reducing the negative impact of local hypoxia on tumor cells, thereby improving the tumor microenvironment and reducing tumor invasiveness (146).

Exercise also plays a critical role in improving the tumor microenvironment by directly modulating angiogenesis (148). Angiogenesis is the formation of new blood vessels, a process that is crucial for tumor growth. Tumor cells secrete angiogenesis-promoting factors, such as VEGF and basic fibroblast growth factor (bFGF), to stimulate blood vessel growth and ensure adequate oxygen and nutrient supply to the tumor. However, tumor blood vessels often have abnormal morphology and loose structures, leading to impaired blood flow and inadequate oxygen and nutrient supply (149). Exercise enhances endothelial cell function, promotes new blood vessel formation, and regulates angiogenesis, thereby optimizing the tumor blood supply and microenvironment, ultimately improving therapeutic outcomes (150).

However, it is important to note that angiogenesis within the tumor microenvironment is not a simple physiological process, and its changes have profound effects on tumor progression, metastasis, and therapy outcomes. Excessive angiogenesis can lead to structurally abnormal tumor blood vessels, resulting in impaired blood flow and creating a vicious cycle that may facilitate tumor cell invasion and metastasis (151). Therefore, exercise not only promotes angiogenesis but also regulates its extent, ensuring balanced angiogenesis that favors treatment (152). In cancer therapy, maintaining moderate angiogenesis improves blood supply to the tumor, enhancing its oxygen and nutrient status while avoiding the negative effects of excessive angiogenesis, such as tumor spread and metastasis (153).

In clinical practice, the application of exercise as an adjunctive treatment is gaining increasing attention (154). Many patients with cancer undergo regular exercise alongside standard treatments to improve their overall health and treatment outcomes. Exercise not only enhances physical strength and immune function but also improves the tumor microenvironment blood supply, thereby enhancing the effectiveness of therapeutic drugs and improving patients’ quality of life (115). However, exercise therapy is not suitable for all patients, particularly those who are physically weak or in the early stages of treatment (155). The intensity and type of exercise should be individualized according to the patient’s physical condition (154). Therefore, exercise should be conducted under the supervision of a professional medical team to ensure safety and effectiveness (156).

In summary, exercise improves the tumor microenvironment by regulating blood supply and angiogenesis in the tumor region, offering new approaches and methods for cancer treatment that warrant further investigation. By enhancing oxygen and nutrient supply to the tumor, exercise not only supports tumor cell metabolism and growth but also enhances the effectiveness of antitumor drugs, improving overall treatment outcomes (110). In future cancer therapies, exercise may become an important adjunctive treatment, providing a more comprehensive therapeutic regimen and improving patients’ quality of life.

### 3.3 Exercise-induced molecular factors

Irisin is a molecule secreted by muscles during physical activity, and as an exercise-induced myokine, it has garnered widespread attention in the scientific community (157). Initially discovered in relation to fat metabolism, irisin promotes the transformation of white fat into brown fat, helping to regulate energy expenditure and metabolic balance (158). However, as research deepened, it became evident that irisin’s functions extend far beyond metabolism alone. Growing evidence suggests that irisin plays a significant role in tumor suppression, immune regulation, and exercise performance (159). Notably, in the field of cancer treatment, irisin, a natural molecule, has demonstrated unique antitumor potential (160). By inducing tumor cell cycle arrest, increasing apoptosis, and inhibiting tumor cell proliferation, irisin provides novel targets and strategies for the treatment of malignant tumors (161).

The antitumor effects of irisin are first reflected in its ability to inhibit tumor cell growth (162). Specifically, irisin upregulates the expression of p21, which inhibits the expression of key cell cycle proteins, such as Cyclin D and Cyclin E, preventing cells from progressing from the G1 phase to the S phase (161). As a cell cycle inhibitor, p21 binds to cyclin-dependent kinases (CDKs), inhibiting CDK activity, leading to cell cycle arrest in the G1 phase, and preventing further tumor cell proliferation (163). This mechanism has been confirmed *in vitro* and in mouse models. By promoting p21 expression, irisin significantly inhibits tumor cell proliferation, slows tumor growth, and enhances the effectiveness of antitumor therapies (164).

In addition to inducing cell cycle arrest, irisin plays an essential role in regulating tumor cell apoptosis (160). Research indicates that irisin activates apoptotic pathways, promoting programmed cell death in tumor cells (165). Irisin enhances oxidative stress within cells, increasing the levels of reactive oxygen species (ROS) (166). Elevated ROS levels lead to damage to proteins, lipids, and DNA, activating apoptotic signaling pathways and ultimately resulting in tumor cell death (25, 167). More importantly, irisin regulates the expression of Bcl-2 family proteins, affecting the balance between pro-apoptotic and anti-apoptotic factors within cells (168). Irisin upregulates pro-apoptotic factors, such as Bax, and downregulates anti-apoptotic factors, such as Bcl-2, thereby activating mitochondrial-mediated apoptosis pathways and promoting tumor cell death (165). Through this mechanism, irisin not only effectively inhibits tumor cell proliferation but also enhances tumor cell death, offering new strategies for cancer treatment (161).

The potential of irisin in cancer therapy extends beyond the regulation of cell-cycle arrest and apoptosis. Recent studies suggest that irisin can also inhibit tumor invasion and metastasis by improving the tumor microenvironment (160). Tumor metastasis, a leading cause of cancer-related deaths, is closely associated with the tumor microenvironment, which provides favorable conditions for tumor cell growth and spread, including hypoxia, acidosis, and immune suppression (169). Irisin modulates immune cell functions in the tumor microenvironment, enhancing the immune system’s ability to clear tumor cells and thereby suppressing tumor metastasis (170). For instance, studies have shown that irisin can regulate the activity of natural killer (NK) cells, enhancing the immune system’s surveillance and clearance of tumor cells and reducing the likelihood of tumor cells invading the blood and lymphatic systems (164). Additionally, irisin improves the structure and function of tumor blood vessels, reducing hypoxia within tumor tissues and thereby inhibiting tumor cell spread (50, 170).Another remarkable effect of irisin in antitumor treatment is its supportive role in enhancing the efficacy of anticancer drugs (171). Research has shown that irisin enhances the permeability of drugs in tumors, helping drugs reach tumor sites more effectively and exert their therapeutic effects (172). Tumor cells often exhibit strong drug resistance, particularly during chemotherapy and radiotherapy, which leads to diminished treatment efficacy. Irisin improves drug delivery by modulating the tumor microenvironment, overcoming drug resistance, and enhancing treatment effectiveness (173). For example, irisin upregulates vascular endothelial growth factor (VEGF), enhancing blood vessel permeability in the tumor region, which facilitates better penetration of antitumor drugs into the tumor tissue (164). Irisin not only increases drug accumulation in tumors but also improves immune cell function in the tumor microenvironment, increasing tumor cell sensitivity to drugs (171).

The antitumor effects of irisin have shown great potential in the treatment of highly malignant tumors, such as glioblastoma multiforme (GBM) (161). GBM is a highly aggressive brain tumor that is notoriously difficult to treat because of the severe limitations posed by the blood-brain barrier (BBB) on drug permeability (174). However, irisin has been found to improve the permeability of the blood-brain barrier, allowing antitumor drugs to penetrate the BBB more effectively and reach the tumor (120). This mechanism positions irisin as a promising new strategy for treating brain tumors, such as glioblastoma, offering better therapeutic prospects for patients (161).

The antitumor effects of irisin are not limited to glioblastoma. Studies have shown that irisin inhibits various types of cancer cells, including breast, lung, and colorectal cancer cells (170). Through its multiple mechanisms of action, irisin not only effectively inhibits tumor cell proliferation but also enhances tumor cell sensitivity to treatment, thereby improving treatment outcomes (172). As a natural molecule, irisin offers good safety and tolerance, making it a promising adjunct to cancer therapy (170).

Although clinical trials directly testing irisin in patients with glioma are lacking, phase I trials in breast cancer (NCT04350463) show that recombinant irisin (100 μg/kg, biweekly IV) is well tolerated and reduces circulating IL-6. Translational strategies for glioma include intranasal delivery to bypass the BBB and CRISPR-activation of FNDC5 (Irisin precursor) in muscle. Beyond irisin, high-throughput sequencing analyses in gene expression regulation studies have identified exercise-induced miR-210 as a novel mediator. This microRNA demonstrates dual oncogenic effects by targeting tumor suppressor genes while activating PI3K/AKT and Wnt/β-catenin signaling pathways, thereby promoting glioma cell proliferation and chemoresistance. These findings reveal potential therapeutic targets underlying the paradoxical effects of exercise intervention (175).

Although the potential of irisin in cancer treatment has been widely studied and validated, its clinical application faces several challenges. Future research should further explore the efficacy of irisin in different cancer types, assess its combined effects with other therapies (such as chemotherapy, radiotherapy, and immunotherapy), and determine optimal treatment regimens. Additionally, the mechanisms of action of irisin require further investigation to reveal its multi-layered effects in cancer treatment (170). These studies lay a solid foundation for the clinical application of irisin and offer new insights and methods for cancer treatment (176).

## 4 Synergistic effects of exercise and drug therapy

### 4.1 Experimental and clinical evidence

#### 4.1.1 Efficacy of combined exercise and chemotherapy in mouse models

**Figure 3:**
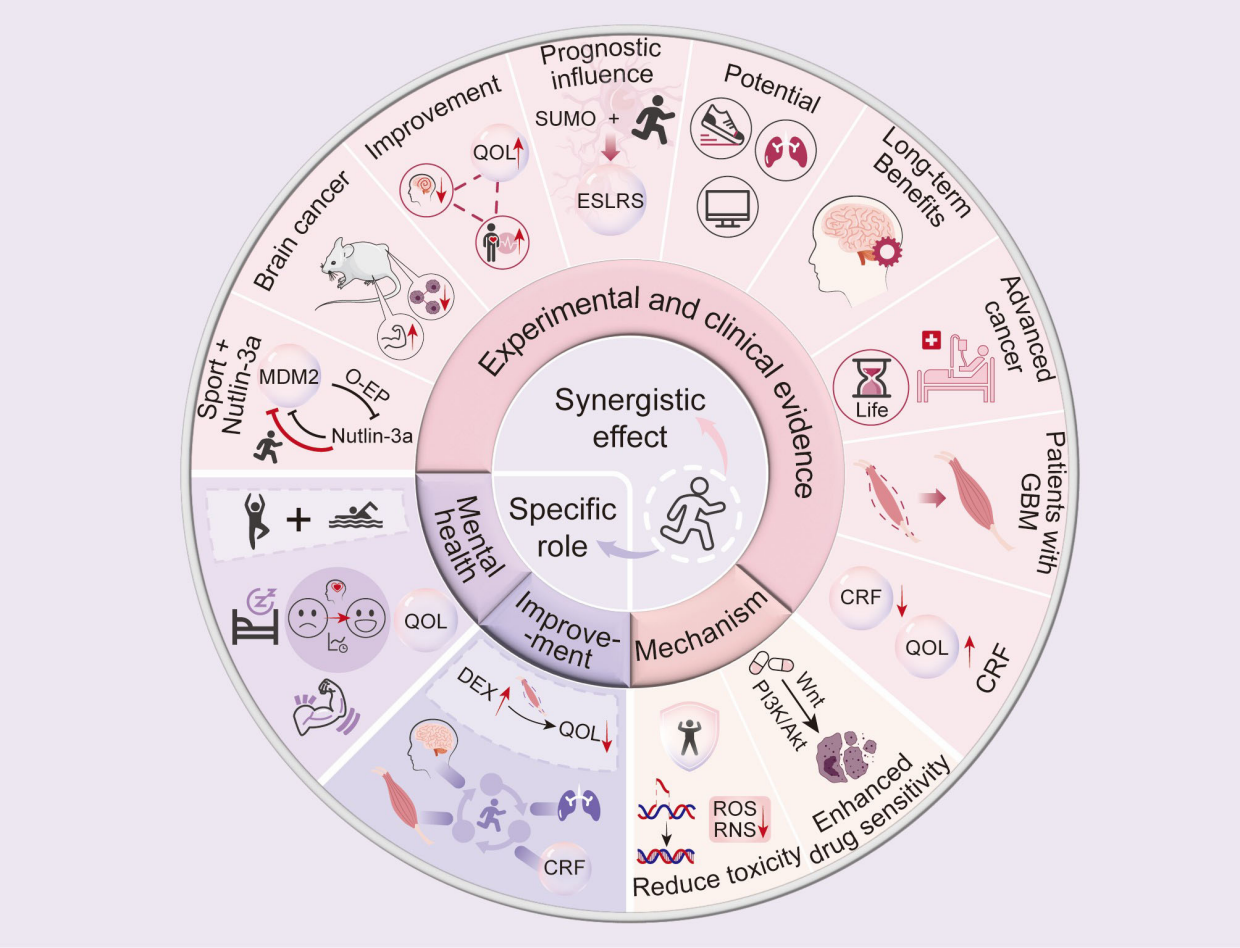
Synergistic Effects of Exercise on Glioblastoma Treatment: Experimental and Clinical Evidence. This figure shows experimental and clinical evidence supporting exercise as a beneficial addition to glioblastoma (GBM) treatment. Exercise enhances quality of life, mental health, and treatment efficacy by modulating biological pathways, reducing drug toxicity, and increasing sensitivity to therapies targeting the MDM2-p53 axis. It affects molecular pathways like Wnt/β-catenin, PI3K/AKT, and oxidative stress regulation, aiding cellular protection and tumor suppression. Exercise can reduce cancer-related fatigue, offer long-term benefits, and improve prognosis. Prognostic markers like SUMO and ESRLS suggest exercise may improve survival outcomes. This emphasizes the importance of integrating exercise into GBM treatment to boost therapeutic efficacy and patient well-being.

Research has shown that the synergistic effect of exercise and drug therapy, particularly with natural compounds, plays a significant role in cancer treatment (113). In mouse model experiments, we found that Nutlin-3a, a natural compound and MDM2 inhibitor, effectively inhibited glioma cell proliferation and activated the p53 pathway (177). The efficacy of Nutlin-3a may be affected by MDM2 overexpression; however, exercise can reverse this effect (177). This was further validated in a mouse model of LGG, where the combination of physical exercise and Nutlin-3a improved the physical function of tumor-bearing mice with MDM2 expression deficiency (20). This finding highlights the synergistic effects of exercise and natural products and reveals their role in immune modulation, suggesting that the combination of exercise and natural compounds may be a new approach for glioma treatment (178) as shown in **Figure 3**.

#### 4.1.2 Exercise and reduction in brain cancer mortality risk

Epidemiological studies have demonstrated that exercise significantly reduces the risk of mortality in patients with brain cancer (179). However, while existing studies emphasize the impact of exercise on brain cancer mortality, the direct relationship between physical exercise and glioma progression remains unclear (180). In an experiment using a high-grade glioma mouse model, we investigated the effect of voluntary physical exercise on tumor proliferation and exercise ability in mice (20). The study found that voluntary exercise significantly reduced the proliferation rate of cortical motor tumors in mice and delayed the onset of motor dysfunction caused by gliomas (20, 25). Thus, physical exercise may serve as an adjunctive intervention in neuro-oncology, helping patients preserve motor function and mitigate the behavioral effects of gliomas.

#### 4.1.3 Impact of exercise interventions on quality of life and treatment outcomes in clinical trials

A systematic review assessed the effects of exercise interventions on the health outcomes of patients with brain cancer. By searching databases such as PubMed and EMBASE, the review identified studies related to brain cancer, and the results indicated that higher levels of physical activity were associated with fewer disease symptoms and better quality of life in patients with brain cancer (180). Preliminary evidence suggests that exercise benefits various aspects, including cancer symptoms, quality of life, and body composition, and has a positive effect on alleviating cancer-related symptoms (181). However, the strength of this evidence remains weak, and high-quality studies are required to confirm these findings.

#### 4.1.4 exercise and prognosis in glioma patients

Recent research has indicated that exercise significantly influences the prognosis of patients with glioma (182). Furthermore, the identification of glioma-specific biomarkers and analysis of molecular pathways enable researchers to predict disease progression and treatment response with enhanced precision (183–185).Exercise not only reduces the risk of mortality but may also promote neuroregeneration (186). In recent years, researchers have focused on the role of small ubiquitin-like modifier (SUMO) proteins in the anticancer effects of exercise and have developed exercise and SUMO-related gene signatures (ESLRS) using machine learning methods (187). This signature reveals how exercise improves the prognosis of low-grade gliomas and other cancers (182).In evaluating treatment efficacy and prognostic indicators, integrating factors such as glioma metabolic profiles and immune cell signatures provides a multidimensional perspective for assessing disease progression and therapeutic responses (188, 189) as shown in **Figure 3**.

#### 4.1.5 Exercise potential in brain tumor treatment

Exercise has the potential to mitigate various health impairments during brain tumor treatment. A systematic review of the impact of exercise on children with brain tumors found that exercise positively affects neuroimaging, physical fitness, and cardiopulmonary function (21). While the effects of exercise on cognition remain unclear, the overall results suggest that exercise interventions can improve physical fitness and quality of life in patients without exacerbating symptoms (190). Therefore, exercise may be an essential component of pediatric brain tumor treatment.

#### 4.1.6 Long-term benefits of exercise for brain tumor survivors

Brain tumor survivors often face a range of complications due to the complexities of the treatment and tumor pathology (14). Research has shown that exercise has a positive effect on the recovery and quality of life of these patients (191). Exercise helps survivors improve cognitive function, enhances motor abilities, and shows improvements in brain structure as detected by magnetic resonance imaging (192, 193). Furthermore, exercise therapy is linked to cognitive performance improvements, particularly in children who have undergone brain tumor treatment, where exercise effectively restores neurocognitive functions (194).

#### 4.1.7 Exercise effects on advanced cancer patients

Patients with advanced cancer often face issues such as fatigue and reduced physical function (19). In recent years, an increasing body of evidence has supported the use of exercise interventions in palliative and end-of-life care (195). Studies have found that over 90% of patients with advanced cancer can undergo exercise therapy (196). Exercise not only improves physical strength but also benefits caregivers (197). Despite some challenges, exercise intervention is considered a feasible and effective approach for treating patients with advanced cancer (198).

#### 4.1.8 Exercise and glioblastoma multiforme patients

Exercise intervention studies in patients with glioblastoma (GBM) have shown that exercise can improve functional performance and quality of life (199). Regular exercise interventions during treatment help patients regain strength, enhance muscle function, and improve their quality of life (200). The results of this study suggest that exercise rehabilitation can play a positive role in the treatment of patients with GBM (201).

#### 4.1.9 Exercise and cancer-related fatigue

Cancer-related fatigue (CRF) is a common symptom among patients with cancer, significantly affecting their quality of life (202). Studies have shown that exercise interventions help alleviate CRF and improve patients’ quality of life (203). Exercise intervention has been shown to relieve fatigue, improve quality of life, and is highly feasible in patients with high-grade gliomas (204). These results suggest that exercise may be an effective intervention for combating cancer-related fatigue (203).

### 4.2 Mechanistic analysis

#### 4.2.1 Exercise enhances drug sensitivity by regulating PI3K/Akt, Wnt pathways

The benefits of exercise in cancer treatment extend beyond improving physical health, as it also enhances drug sensitivity by regulating intracellular signaling pathways (106). Studies have found that exercise can modulate tumor cell growth and differentiation through pathways such as PI3K/Akt and Wnt, thereby increasing the cytotoxic effect of drugs on tumor cells (110). Integrative metabolomics and bioinformatics analyses in cellular metabolism studies reveal progesterone’s therapeutic enhancement mechanism. The hormone potentiates antiglioma drug efficacy through AMPK/mTOR pathway modulation, establishing novel pharmacological optimization strategies (205). Furthermore, innovative nanodrug delivery systems, such as near-infrared light-activated upconversion nanoparticle/curcumin hybrid formulations, have demonstrated significant therapeutic potential by inducing differentiation and elimination of glioma stem cells (206). This combinatorial approach may offer enhanced treatment efficacy via multitargeted regulatory mechanisms, thereby presenting novel avenues for precision glioma therapy.

#### 4.2.2 Mechanisms of reducing treatment-related toxicity

Cancer treatments, particularly chemotherapy and radiotherapy, are often associated with severe side effects (207). Exercise reduces treatment-related toxicity through several mechanisms, such as decreasing oxidative stress, enhancing immune function, and repairing DNA damage (208). These effects not only improve the patient’s quality of life but also enhance overall treatment efficacy (113). Therefore, exercise can be an effective adjunctive therapy in cancer treatment, helping to mitigate the adverse effects of drugs and treatments (110).

#### 4.2.3 Summary

Overall, the combination of exercise and drug therapy offers a new treatment strategy for patients with brain tumors. Experimental and clinical studies have shown that exercise can not only improve patients’ physiological functions but also enhance treatment outcomes by regulating cellular signaling pathways, increasing drug sensitivity, and reducing treatment-related toxicity (113, 209, 210). Future research should continue to explore the synergistic effects of exercise interventions and drug therapy and develop personalized exercise treatment plans to maximize their clinical application.

## 5 Specific effects of exercise on glioma patients

Exercise has a broad and profound impact on patients with glioma, influencing physical function, quality of life, psychological health, and other aspects. According to current research, exercise can not only enhance patients’ physical and cognitive abilities but also significantly improve their mental health, alleviate fatigue, reduce anxiety and depression, and improve treatment adherence and quality of life (211). The following is a detailed exploration of the specific effects of exercise interventions on patients with glioma.

### 5.1 Improvement of physical function and quality of life

Enhancing physical endurance and alleviating fatigue are among the most direct benefits of exercise in patients with glioma (204). Long-term or high-dose use of steroid drugs, such as dexamethasone (DEX), leads to muscle atrophy in 10%-60% of patients with glioblastoma, significantly affecting their physical function and quality of life (QOL) (212). Consequently, an increasing number of studies support exercise as an effective adjunctive therapy to help improve functional capacity and reduce treatment-related side effects. Particularly in the context of resistance training, research has shown that such training can increase muscle mass, strength, and functional fitness in older adults and certain cancer patients (213). Although research on exercise interventions in patients with glioblastoma is relatively limited, preliminary evidence suggests that exercise is safe and feasible (180). For example, a systematic review of exercise interventions in childhood cancer survivors (CCS) who had completed anti-cancer treatment at least one year prior indicated that, despite low methodological quality, early evidence suggests that exercise interventions could improve brain volume and structure in childhood brain tumor survivors (21).

Additionally, patients with glioma often experience cognitive impairments, which severely impact their quality of life and interfere with daily life, social, and professional activities (214). Increasing evidence shows that exercise promotes experience-dependent brain plasticity, which helps in the structural and functional recovery of the brain following damage (215). For instance, a randomized controlled trial (RCT) in patients with glioma demonstrated that exercise intervention helped improve cognitive functions, including attention, information processing speed, verbal memory, and executive function (201). Moreover, exercise interventions significantly improved self-reported fatigue, mood, sleep quality, and health-related quality of life (216). Specifically, during a six-month intervention, the exercise group outperformed the control group in various cognitive tests, although the exercise group showed slightly poorer results in sustained selective attention (217). Furthermore, exercise interventions have been shown to improve neurocognitive function, body composition, and motor ability (218). In exercise studies on other cancer patients, aerobic and resistance training have been proven to enhance muscle strength, endurance, and aerobic capacity (156). Studies on patients with glioma also support this conclusion, showing that even small-sample trials can yield positive clinical effects (219).

### 5.2 Psychological health effects

Exercise also has a significant positive impact on the psychological health of patients with glioma (192). Several studies have shown that exercise interventions can effectively reduce negative emotions, such as anxiety and depression, and improve patients’ psychological health (220, 221). A study on a novel independent home exercise program found that patients with glioma who engaged in exercise generally demonstrated better adherence and improved quality of life (222). In this study, nine out of 14 participants (60%) adhered to the exercise regimen for a month. Patients who exercised more frequently tended to have higher marital satisfaction and income levels and showed positive trends in quality-of-life scores. Another study reported the effects of a 12-week exercise intervention involving two patients with glioma (192). The participants completed biweekly 1-hour aerobic and resistance-training sessions. At the 6- and 12-week assessments, the patients showed improvements in strength, cardiovascular health, and psychological well-being (e.g., reduced depression and anxiety and improved quality of life). In particular, patients generally experienced a reduction in psychological distress in self-reported anxiety and depression. Additionally, a randomized controlled trial on patients with high-grade glioma investigated the effects of endurance and resistance training on psychological health, sleep quality, and quality of life (223). The results showed that patients who received exercise interventions showed significant improvements in physical strength, sleep quality, and anxiety symptoms. Compared with the control group, the exercise intervention group demonstrated more substantial improvements in psychological health and sleep quality. Furthermore, combining aerobic exercise with flexibility training has been shown to have significant effects on both psychological health and physiological function in patients with glioma (192). In one study, a female patient who underwent 36 sessions of aerobic and flexibility training experienced a 20% reduction in fatigue and nearly a 70% improvement in quality of life (224). However, despite the positive effects on psychological and physiological health, improvements in cognitive function require further investigation. Overall, although research on exercise interventions in patients with glioma is relatively sparse, existing evidence suggests that exercise not only improves physical function and cognitive abilities but also enhances psychological health, reduces anxiety and depression, and improves treatment adherence and quality of life (225). Therefore, exercise holds significant clinical and research value as an adjunctive therapy for patients with glioma.

### 5.3 Conclusion and outlook

In conclusion, exercise interventions for patients with glioma not only improve physical health but also positively affect psychological health, quality of life, and cognitive function (201). Although existing research provides preliminary evidence, many questions remain unanswered. For instance, how to design more personalized exercise programs and how to quantify the long-term benefits of exercise for patients with glioma. Addressing these issues will provide more scientific evidence for glioma treatment and promote the widespread clinical application of exercise interventions in this context.

Future research should further explore the mechanisms underlying exercise interventions in patients with glioma and investigate the optimal timing, intensity, and frequency of different types of exercise (201). This will allow for more effective and personalized treatment plans for patients. Moreover, based on the broad benefits of exercise interventions for patients with glioma, exercise therapy is likely to become a standard adjunctive treatment, offering comprehensive treatment support.

## 6 Research status and limitations

### 6.1 Limitations of current research

**Figure 4:**
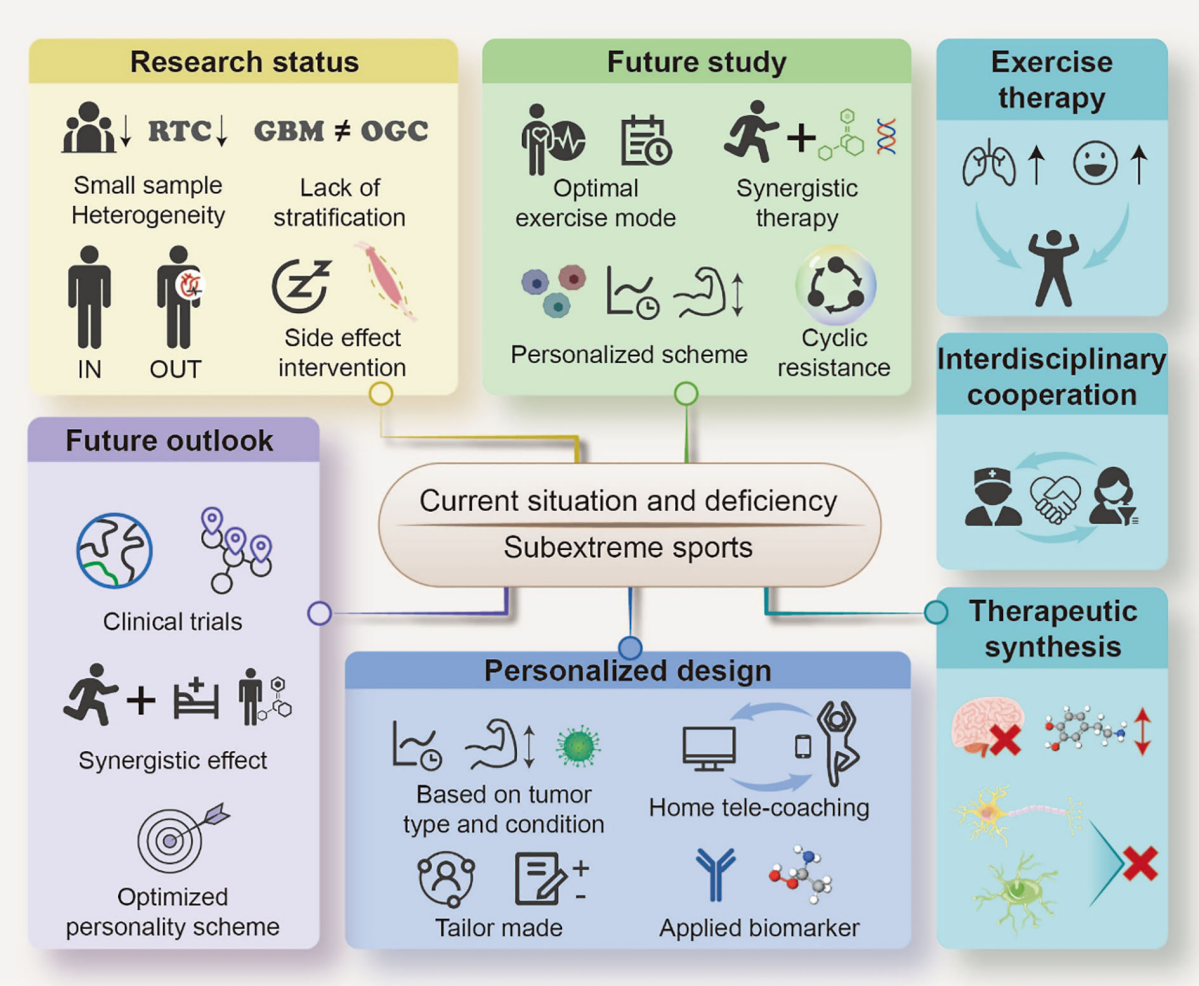
Current Situation, Deficiencies, and Future Directions of Exercise Therapy for Glioma Treatment. This figure summarizes the existing challenges, research status, and future perspectives of exercise therapy as an adjunctive treatment for gliomas. The current situation and deficiencies highlight the need to refine exercise-based interventions for cancer care. The research status section outlines key limitations, including small sample sizes, heterogeneity, lack of stratification between GBM and other glioma cancers (OGC), and the necessity for intervention for side effects. The future study section emphasizes the need to optimize exercise modes, implement synergistic therapy, and utilize cyclic resistance training, with a focus on personalized treatment schemes. The future outlook discusses the potential of clinical trials, synergistic effects of exercise and therapy, and development of optimized personality schemes for patient-centered care. The personalized design section highlights the importance of tailoring exercise regimens based on tumor type and patient condition, incorporating home tele-coaching, and applying biomarkers to improve the treatment efficacy. Furthermore, exercise therapy is linked to interdisciplinary cooperation and therapeutic synthesis, advocating integrated strategies that enhance patient outcomes and mitigate treatment-related side effects. This framework underscores the necessity for continued research and clinical application of exercise-based interventions for glioma management.

Despite the broad positive impact of exercise on cancer patients’ rehabilitation, research specifically focused on patients with brain tumors, particularly those with high-grade glioma (HGG), remains insufficient as shown in **Figure 4**.

#### 6.1.1 Limited sample size and heterogeneity in research methods

Although multiple studies have been conducted on exercise interventions, research specifically targeting patients with brain tumors, particularly those with high-grade gliomas, remains limited (180). Existing studies often face challenges of small sample sizes and a lack of consistency in study design, making it difficult to draw broadly applicable conclusions (180). For example, physical fitness, cardiopulmonary function, muscle mass, and strength in brain tumor patients are often affected during treatment. However, exercise intervention studies for this group are still less abundant compared to other cancer populations (Ballard-Barbash et al., 2012; Singh et al., 2013; Buffart et al., 2014) (192). The heterogeneity of these studies is primarily manifested in differences in research methods and data collection, including the absence of systematic randomized controlled trials (RCTs).

#### 6.1.2 Lack of stratified research on different tumor subtypes

There has been insufficient comparative research on the effects of exercise interventions in patients with different types of brain tumors, particularly low-grade and high-grade gliomas (226). Each tumor type has distinct biological characteristics; therefore, there may be significant differences in the effects of exercise interventions (227). However, most current studies do not conduct stratified analyses by tumor subtype or offer detailed intervention strategies or effect comparisons. For example, patients with glioblastoma (GBM) may have different exercise tolerance and outcomes compared to patients with oligodendrogliomas; however, this aspect has not been fully explored in the existing literature.

#### 6.1.3 Limitations in research methods

Most current research on exercise interventions for brain tumor survivors is observational, with small sample sizes and relatively high attrition rates (228). Since patients with brain tumors often have comorbidities (e.g., heart disease, neurological deficits), these patients are often excluded from studies, leading to recruitment bias (229). This bias is particularly significant in patients with brain cancer, as only those in relatively good health are likely to participate in exercise intervention studies.

#### 6.1.4 Recruitment and retention issues in exercise interventions

Despite the potential benefits of exercise interventions, recruitment and retention remain major challenges in brain tumor research (228). Since brain tumor treatment often involves multiple side effects (e.g., fatigue, cognitive impairment, and muscle wasting), these side effects may affect patients’ willingness to participate and their adherence to interventions (230). This makes it more complex to conduct exercise intervention studies and difficult to generalize the findings to a broader patient population.

### 6.2 Future research directions

#### 6.2.1 Investigating the optimal type, intensity, and frequency of exercise

Future research should further explore the effects of different types of exercise interventions (e.g., aerobic exercise, resistance training) and the optimal intensity and frequency for patients with brain tumors, particularly those with high-grade gliomas. Although existing studies have shown the benefits of aerobic and resistance training for individuals with cancer, most of these studies have focused on other cancer populations, and the specific needs and intervention effects for individuals with brain tumors have not been thoroughly studied (192). Therefore, future studies should systematically analyze the specific effects of different exercise programs on patients with brain tumors and identify the most suitable exercise types and intervention intensities.

#### 6.2.2 In-depth study on the synergistic effects of exercise and emerging therapies

Emerging therapies, such as immunotherapy and gene therapy, have become important directions in glioma treatment (231). Future research should investigate the synergistic effects of exercise in combination with these emerging therapies. Novel targeted therapeutic strategies are emerging as potential game-changers, offering the dual benefits of enhanced treatment efficacy and minimized side effects while advancing precision medicine frontiers (232). It is worth exploring whether exercise can enhance the effects of immunotherapy or gene therapy or whether it can alleviate the side effects of these treatments (91). Research on this synergistic effect could lead to more personalized and comprehensive treatment plans for patients (233, 234).

#### 6.2.3 Development of personalized exercise intervention programs

Given the significant differences in pathology, treatment responses, and side effects among patients with brain tumors, future research should focus on developing personalized exercise intervention programs (154). Studies have tailored exercise regimens to individual patients based on factors such as cancer type, treatment stage, and physical fitness level (235). Personalized interventions not only increase patient adherence but also maximize the effectiveness of exercise in improving functional capacity, reducing fatigue, and enhancing quality of life (236).

#### 6.2.4 Verifying the effectiveness of circuit resistance training for glioblastoma patients

Existing research has shown that personalized circuit resistance training can improve functional fitness in patients with cancer and may help mitigate steroid-induced myopathy (237). Studies on patients with glioblastoma have also indicated that personalized exercise interventions can effectively enhance physical fitness and overall quality of life (192, 204). Future studies should further verify these findings and explore the applicability and effectiveness of circuit resistance training in patients with different types of brain tumors. Future research should also consider factors such as patients’ lifestyle, disease activity, and the feasibility of exercise interventions.

## 7 Exploring the safety and efficacy of submaximal exercise for glioma patients

Submaximal exercise refers to physical activities that do not exceed a patient’s maximum heart rate but effectively activate cardiovascular function and muscle strength (238). A program named ActiNO has demonstrated that submaximal exercise is both safe and effective in patients with glioma (239). Future research should focus on exploring the long-term effects of this exercise regimen, particularly its potential to extend survival and improve quality of life. These studies could help in the development of more targeted exercise programs tailored to patients with brain tumors.

Enhancing Quality of Life——To achieve this, exercise therapy should be incorporated into the comprehensive treatment plan for glioma, becoming a routine part of care.

### 7.1 Incorporating exercise therapy into glioma comprehensive treatment guidelines

Currently, the primary treatments for gliomas rely on surgery, radiotherapy, and chemotherapy (240). However, these therapies often come with significant side effects, such as cognitive decline, fatigue, and motor dysfunction, which severely affect the patient’s daily life and quality of life (41). Therefore, including exercise therapy in the treatment guidelines is crucial for improving recovery outcomes. Studies have found that exercise not only alleviates fatigue but also enhances muscle strength, boosts cardiovascular endurance, and improves cognitive function, all of which contribute to better overall health (241).

### 7.2 Multidisciplinary team collaboration

Treatment for patients with glioma requires a multidisciplinary team, typically consisting of oncologists, rehabilitation specialists, exercise medicine experts, nutritionists, and psychologists. This collaborative approach ensures comprehensive treatment, addressing the patient’s physical, psychological, and social recovery needs (242). Based on the patient’s specific requirements, the team can create personalized treatment plans and adjust them according to the patient’s responses. This integrated treatment model allows patients to effectively cope with the side effects of tumor treatment while improving their quality of life and functional independence during the process (243).

### 7.3 Enhancing the comprehensiveness of rehabilitation treatment

Patients with glioma often experience a variety of sequelae, such as cognitive impairments, fatigue, and visual perception changes, which severely impact their quality of life (214). A more comprehensive rehabilitation model is urgently needed to address these issues. Patient recovery should not be limited to directly addressing the effects of tumor treatment but should also consider interventions for various complications, including cognitive impairment, emotional fluctuations, and motor dysfunction (244, 245). With a more refined rehabilitation model, it is possible to effectively slow the functional decline of patients and help them regain the ability to perform daily activities. The advantages of multidisciplinary collaboration lie in the ability to jointly assess and intervene, continuously identifying and addressing functional limitations (246).

Research has shown that early multidisciplinary rehabilitation interventions can significantly reduce disability rates and improve daily living abilities in patients with glioma. For example, in a study involving patients with brain tumors, after 12 weeks of rehabilitation intervention, patients showed significant improvements in physical function scores, cognitive function, and social functioning (P < 0. 0001) (247). This demonstrates the effectiveness of early multidisciplinary rehabilitation in reducing symptoms and improving the quality of life.

### 7.4 Personalized program design

#### 7.4.1 Exercise interventions based on tumor type and disease status

Each patient with glioma has unique circumstances, including disease stage, physical condition, treatment responses, and side effects. Therefore, exercise interventions must be personalized to suit individuals. This involves selecting the appropriate types of exercise (such as aerobic exercise and resistance training), intensity, and frequency, as well as considering the patient’s disease stage, functional level, and the toxicity of their treatments. For example, some patients may experience significant physical decline due to the tumor or treatment side effects, whereas others may have better physical capacity and tolerate higher-intensity interventions. Personalized exercise prescriptions can ensure that patients maximize the benefits of exercise while ensuring safety (248).

#### 7.4.2 Feasibility of home-based remote exercise interventions

A pilot study involving patients with glioma explored the feasibility of home-based remote exercise interventions (222). The study, designed as a randomized controlled trial (RCT), involved stable grade II and III glioma patients who underwent a six-month intervention. The patients exercised at home three times per week, with the intensity set at 60%-85% of their maximum heart rate. Heart rate monitors were worn by the patients, and data were monitored and feedback was provided through an online platform. The results of this study showed that home-based remote interventions were feasible for a small group of willing participants and could significantly improve cardiovascular function, physical activity, and body mass index. This further supports the need for large-scale exercise intervention trials in patients with glioma.

#### 7.4.3 Tailored exercise prescriptions

Patients with glioma often experience fluctuating disease stages and various side effects from treatment; therefore, exercise interventions need to be flexibly adjusted based on individual conditions (199). Exercise prescriptions should be designed according to factors such as the patient’s physical condition, treatment toxicity, and disease progression. For example, some patients may be too weak post-treatment to engage in high-intensity exercise, whereas others may tolerate higher exercise volumes. Tailored exercise prescriptions can maximize physical strength and quality of life and minimize the risk of injury or adverse reactions associated with exercise.

#### 7.4.4 Application of biomarkers in exercise interventions

To monitor the effects of exercise interventions more precisely, future research should develop biomarkers to evaluate the therapeutic impact of exercise. These biomarkers reflect the physiological state, immune function, and other health indicators influenced by exercise, providing objective data support (249). For example, certain immune system biomarkers or blood metabolic products may partially reflect the physiological regulation induced by exercise (250). Thus, combining biomarker monitoring with exercise could help assess the short-term effects and predict the long-term outcomes of interventions (53).

### 7.5 Challenges in exercise adherence

Although exercise interventions offer significant benefits for patients with glioma, adherence to exercise programs remains a major challenge. Several factors contribute to low adherence rates, including physical limitations, psychological distress, and lack of motivation owing to the severity of the disease. Patients often experience fatigue, pain, and cognitive impairment, which can hinder their ability to engage in regular physical activity (197). Additionally, psychological factors, such as depression, anxiety, and fear of treatment side effects, can negatively affect adherence to exercise protocols (198).

To address these challenges, solutions must focus on enhancing patient motivation and providing support to overcome physical and emotional barriers. For instance, interventions involving regular monitoring, personalized support, and motivational strategies, such as goal-setting or feedback mechanisms, have been shown to improve adherence in cancer populations (199). Implementing supervised exercise sessions or combining physical activity with psychological support (e.g., counseling or group exercises) may also increase participation rates. Additionally, the use of digital platforms, such as mobile apps or online exercise programs, could offer more flexible and accessible ways to support exercise adherence in patients with gliomas (200).

Moreover, clinicians should emphasize the importance of exercise as an integral part of the treatment regimen and address any concerns that patients may have regarding safety and feasibility. Educating patients on the benefits of exercise for symptom management and improving their quality of life can help reduce their resistance to participating in exercise interventions. By addressing these barriers, exercise interventions can become a more effective and sustainable adjunctive therapy for patients with glioma.

### 7.6 Future research directions and challenges

#### 7.6.1 Large-scale, multicenter clinical trials

Owing to the low incidence of brain cancer, single-center studies may struggle to recruit sufficient patient samples. Therefore, large-scale, multicenter international cooperative trials are needed to ensure that the findings are statistically significant and applicable across diverse populations (251). These studies will help validate the effectiveness of exercise interventions in patients with different types of brain tumors.

#### 7.6.2 Further exploration of the synergistic effects between exercise and other treatments

Although research has shown that exercise can improve physical function and quality of life in patients with glioma, the synergistic effects of exercise with other treatments (such as radiotherapy, chemotherapy, and immunotherapy) require further investigation (154, 252). Exploring these interactions could provide more comprehensive treatment strategies, thereby improving the overall treatment efficacy.

#### 7.6.3 Optimization and application of personalized exercise programs

With the development of precision medicine, personalized exercise interventions have become a key component of glioma treatment (253). Future research should focus on optimizing exercise prescriptions and developing more refined and individualized exercise plans. In particular, in the rehabilitation of patients with brain tumors, precise interventions based on functional status, treatment responses, and lifestyle will be an important direction for future research (254, 255).

## 8 Conclusion

Gliomas, particularly glioblastoma multiforme (GBM), are highly aggressive brain tumors that present numerous treatment challenges. Although current therapies, such as radiotherapy, chemotherapy, and targeted treatment, have extended patient survival to some extent, these therapies have limited efficacy and are often accompanied by significant side effects. The biological characteristics of gliomas, including blood-brain barrier permeability, tumor cell heterogeneity, and an immunosuppressive microenvironment, make treatment even more difficult. Moreover, glioblastoma often leads to long-term side effects, such as cognitive dysfunction and neurological decline, which greatly affect the patients’ quality of life. As the incidence of glioma rises globally, particularly with age, improving patients’ quality of life and prolonging survival have become important issues in brain tumor research. In this context, exercise as an adjunctive treatment has gained increasing attention. Exercise not only helps improve physical function and cognitive abilities but may also enhance the patient’s antitumor capacity by regulating the immune system and suppressing inflammation (16–18). Studies have shown that appropriate exercise can significantly reduce the mortality risk in patients with brain cancer and positively influence glioma treatment outcomes.

Exercise interventions in glioma treatment work through several key mechanisms. Firstly, exercise boosts the immune system by activating NK cells and T cells, enhancing immune surveillance and aiding in tumor cell elimination. Secondly, it reduces inflammation in the tumor microenvironment by lowering pro-inflammatory cytokines, helping to slow glioma growth and spread. Additionally, exercise improves blood-brain barrier permeability, increasing the delivery and effectiveness of anti-tumor drugs. Exercise-induced factors, like irisin, may also inhibit tumor cell growth and invasion. Studies show that combining exercise with drug therapies, such as chemotherapy and immunotherapy, improves drug efficacy, reduces side effects, and enhances patients’ quality of life. Though the precise mechanisms are not fully clear, early evidence highlights exercise as a promising adjunctive treatment, with potential to improve survival and prognosis in glioma patients. Further research could solidify its role in future clinical glioma management.
